# Clinical pathologies of bone fracture modelled in zebrafish

**DOI:** 10.1242/dmm.037630

**Published:** 2019-09-03

**Authors:** Monika J. Tomecka, Lalith P. Ethiraj, Luis M. Sánchez, Henry H. Roehl, Tom J. Carney

**Affiliations:** 1 Institute of Molecular and Cell Biology (IMCB), A*STAR (Agency for Science, Technology and Research), 61 Biopolis Drive, Proteos, 138673, Singapore; 2 Department of Biomedical Science, Firth Court, Western Bank, The University of Sheffield, Sheffield, S10 2TN, United Kingdom; 3 Lee Kong Chian School of Medicine, Experimental Medicine Building, Yunnan Garden Campus, 59 Nanyang Drive, Nanyang Technological University 636921, Singapore

**Keywords:** Fracture, Bone, Zebrafish, Osteogenesis imperfecta, Bisphosphonate, Callus, *Staphylococcus aureus*

## Abstract

Reduced bone quality or mineral density predict susceptibility to fracture and also attenuate subsequent repair. Bone regrowth is also compromised by bacterial infection, which exacerbates fracture site inflammation. Because of the cellular complexity of fracture repair, as well as genetic and environmental influences, there is a need for models that permit visualisation of the fracture repair process under clinically relevant conditions. To characterise the process of fracture repair in zebrafish, we employed a crush fracture of fin rays, coupled with histological and transgenic labelling of cellular responses; the results demonstrate a strong similarity to the phased response in humans. We applied our analysis to a zebrafish model of osteogenesis imperfecta (OI), which shows reduced bone quality, spontaneous fractures and propensity for non-unions. We found deficiencies in the formation of a bone callus during fracture repair in our OI model and showed that clinically employed antiresorptive bisphosphonates can reduce spontaneous fractures in OI fish and also measurably reduce fracture callus remodelling in wild-type fish. The *csf1ra* mutant, which has reduced osteoclast numbers, also showed reduced callus remodelling. Exposure to excessive bisphosphonate, however, disrupted callus repair. Intriguingly, neutrophils initially colonised the fracture site, but were later completely excluded. However, when fractures were infected with *Staphylococcus aureus*, neutrophils were retained and compromised repair. This work elevates the zebrafish bone fracture model and indicates its utility in assessing conditions of relevance to an orthopaedic setting with medium throughput.

This article has an associated First Person interview with the first author of the paper.

## Abstract (precis)

**Summary:** The effect of osteogenesis imperfecta, bisphosphonate treatment and bacterial infection on phases of bone fracture repair are determined using a zebrafish fracture model.

## INTRODUCTION

Bone fractures are repaired through sequential phased tissue responses, which are orchestrated and interdependent. These correspond to immediate and long-term requirements at the damage site (Hall, 2015; Schindeler et al., 2008). Initial blood clotting is followed by rapid recruitment of inflammatory cells to remove debris and combat infection. Immigrating chondrocytes and, to a lesser extent, fibroblasts, lay down a soft temporary fibrocartilaginous callus that rapidly stabilises the lesion (Echeverri et al., 2015; Schindeler et al., 2008), but is gradually replaced by a hard bone callus through recruitment of osteoblast precursors. Finally, the new bone is refined and the bone callus is remodelled through recruitment and coupled activity of osteoclasts, the activity of which reduces the callus volume towards the original bone width and leads to mature bone (Li et al., 1999).

Osteomyelitis, age, poor diet or inherited genetic diseases can all reduce bone density or quality and lead to heightened fracture susceptibility, which can be debilitating and life-shortening (Cauley, 2017; Tsuchiya et al., 2017). Osteogenesis imperfecta (OI) is a rare inherited syndrome, mostly caused by mutations in collagen I or enzymes required for its maturation (Rauch and Glorieux, 2004). Most OI patients suffer from spontaneous fractures occurring from childhood or, in severe cases, *in utero*, which is mostly lethal perinatally. Defects in fracture repair of OI patients is also apparent, with variable reports of non-union fractures, where the bone elements fail to re-fuse and the fracture site retains only a fibrocartilage covering (pseudoarthrosis) that fails to progress to a hard callus. Frequency of non-unions in OI varies with severity, with about one-fifth or more of fractures being non-union in some cohorts (Agarwal and Joseph, 2005; Gamble et al., 1988) and up to 64% of pseudoarthroses in severe type III OI, indicative of common non-union (Vetter et al., 1992). In contrast, some OI types (especially type V) show hyperplastic hard callus formation, although this is rare and the underlying mechanism is unclear (Cheung et al., 2007; Ramirez et al., 2003).

In addition to surgical correction, treatment for OI consists largely of anti-resorptives such as bisphosphonates, which inhibit osteoclast activity and thus increase bone mass (Esposito and Plotkin, 2008; Rauch and Glorieux, 2004). Bisphosphonates are absorbed strongly onto bone where they are consumed by osteoclasts, disrupt cell function and, hence, lead to reduced bone resorption with gradual improvements in bone mineral density (Weinstein et al., 2009). Bisphosphonates have become a drug of choice for OI, as well as for osteoporosis, and improve numerous bone parameters in paediatric patients, including bone mass, density, DEXA scores, bone marker biochemistry (both serum and urine), cortical thickness and bone architecture, culminating in reduced spontaneous bone fractures (Astrom and Soderhall, 2002; Drake et al., 2008; Glorieux, 2007; Land et al., 2006; Rauch et al., 2003). In the clinic, the regime, concentration and length of treatment with bisphosphonates varies widely, as does the outcome. Despite widespread use, bisphosphonate trials in OI are small scale and the conclusions often conflict. Whether therapy improves OI patient outcome in the long term, and for which clinical parameter, is currently debated and lacks long-term studies of sufficient power (Bachrach and Ward, 2009). Thus, there is a pressing need for animal OI models that permit quantitative assessment of bisphosphonate effects on fracture rates and healing. Two mouse OI models *oim/oim* (Chipman et al., 1993) and Brtl/+ (Forlino et al., 1999), caused by mutations in *Col1a2* and *Col1a1* genes, respectively, are widely used. Both respond to alendronate, which increases the bone mineral density and structural parameters of the mutant mice, thus reducing fracture rate and resistance to fracture (McCarthy et al., 2002; Uveges et al., 2009).

Although there is good evidence that bisphosphonates reduce fracture occurrence in patients, the effect of these bisphosphonates on the subsequent fracture healing process is less documented. A number of studies in murine fracture models have concluded that administration of bisphosphonate does reduce remodelling, leading to a larger, persistent hard callus that favours healing (Amanat et al., 2005; Koivukangas et al., 2003; Li et al., 1999). In addition, short-term bisphosphonate usage increases fracture callus mineralisation (Adolphson et al., 2000). There are, however, some concerns that extended use of bisphosphonates supresses bone turnover and has detrimental effects on the biomechanical properties of the callus because of the importance of osteoclast remodelling (Kates and Ackert-Bicknell, 2016; Odvina et al., 2005). Extended administration might compromise callus strength (McDonald et al., 2008), and high doses impair repair of stress fractures in the rat (Kidd et al., 2011).

Bacterial infection of the bone (osteomyelitis) is a further factor that reduces bone strength and impairs fracture repair (Lew and Waldvogel, 2004). Most commonly caused by *Staphylococcus aureus*, infection can be the result of bone trauma, implants, surgery or introduced from the vasculature (Carek et al., 2001). *S. aureus* has strong affinity for bone extracellular matrix components (Elasri et al., 2002) and can be internalised into osteoblasts where it can initiate inflammation yet evade lysosomal degradation (Josse et al., 2015). This leads to compromised osteoblast proliferation, differentiation, activity and survival (Sanchez et al., 2013), but also to activation of osteoclasts (Josse et al., 2015; Trouillet-Assant et al., 2015). Together, these factors result in dead bone, decreased bone density and strength (Lew and Waldvogel, 2004; Mouzopoulos et al., 2011), increased fracture risk (Belthur et al., 2012) and impaired fracture healing (Blanchette et al., 2017; Santolini et al., 2015).

A number of species have been used to model implant- and fracture-associated infection models as well as haematological osteomyelitis, including rabbits (Andriole et al., 1973), rats (Rissing et al., 1985), mice (Chadha et al., 1999), sheep (Kaarsemaker et al., 1997), chicks (Emslie and Nade, 1983) and dogs (Fitzgerald, 1983). These models are crucial for understanding the parameters influencing the outcome of clinically heterogeneous bone infection and for testing treatment paradigms.

The use of zebrafish as a system for bone research is nascent yet growing (Mackay et al., 2013), as indicated by the availability of a number of markers, transgenic lines and mutant lines affecting the skeletal system, including models of OI (Asharani et al., 2012; Fisher et al., 2003). The *frilly fins* (*frf*) mutant, for example, corresponds to mutations in *bmp1a* (encoding a metalloprotease required for collagen I maturation) and reflects many features of OI type XIII. Both *frf* and OI type XIII are the result of mutations in the gene encoding BMP1 (Bmp1a in *frf*) (Asharani et al., 2012; Cho et al., 2015; Martinez-Glez et al., 2011). Patients with this form of OI respond to bisphosphonate treatment. It is known that teleost fish possess both osteoclasts and osteoblasts (Spoorendonk et al., 2010) and that bisphosphonates can reduce the activity of osteoclasts in medaka fish (Yu et al., 2016) Additionally, bone fracture models have been reported that have indicated similarities to mammalian fracture repair (Sousa et al., 2012) and identified proximal mature osteoblasts as contributing to fracture repair following dedifferentiation (Geurtzen et al., 2014).

Here, we develop zebrafish as a model for fracture with clinically relevant genetic, pharmacological and bacterial perturbations for comparison of fracture repair parameters. We demonstrate the utility of the zebrafish fracture model for complex and extended analysis of treatment of fracture complications, bringing new assays, enhanced imaging and quantitation to the field.

## RESULTS

### Phases of fracture repair in zebrafish

We developed a zebrafish bone crush fracture model to permit comparison of the phases of repair under clinically relevant scenarios. Using forceps, we crushed four lepidotrichia of an adult zebrafish tail fin (Movie 1) and then applied histological and cellular imaging at different stages to define the temporal phases of bone repair in wild-type (WT) fish, focussing on inflammation, chondrogenesis, ossification and remodelling.

**Fig. 1.**
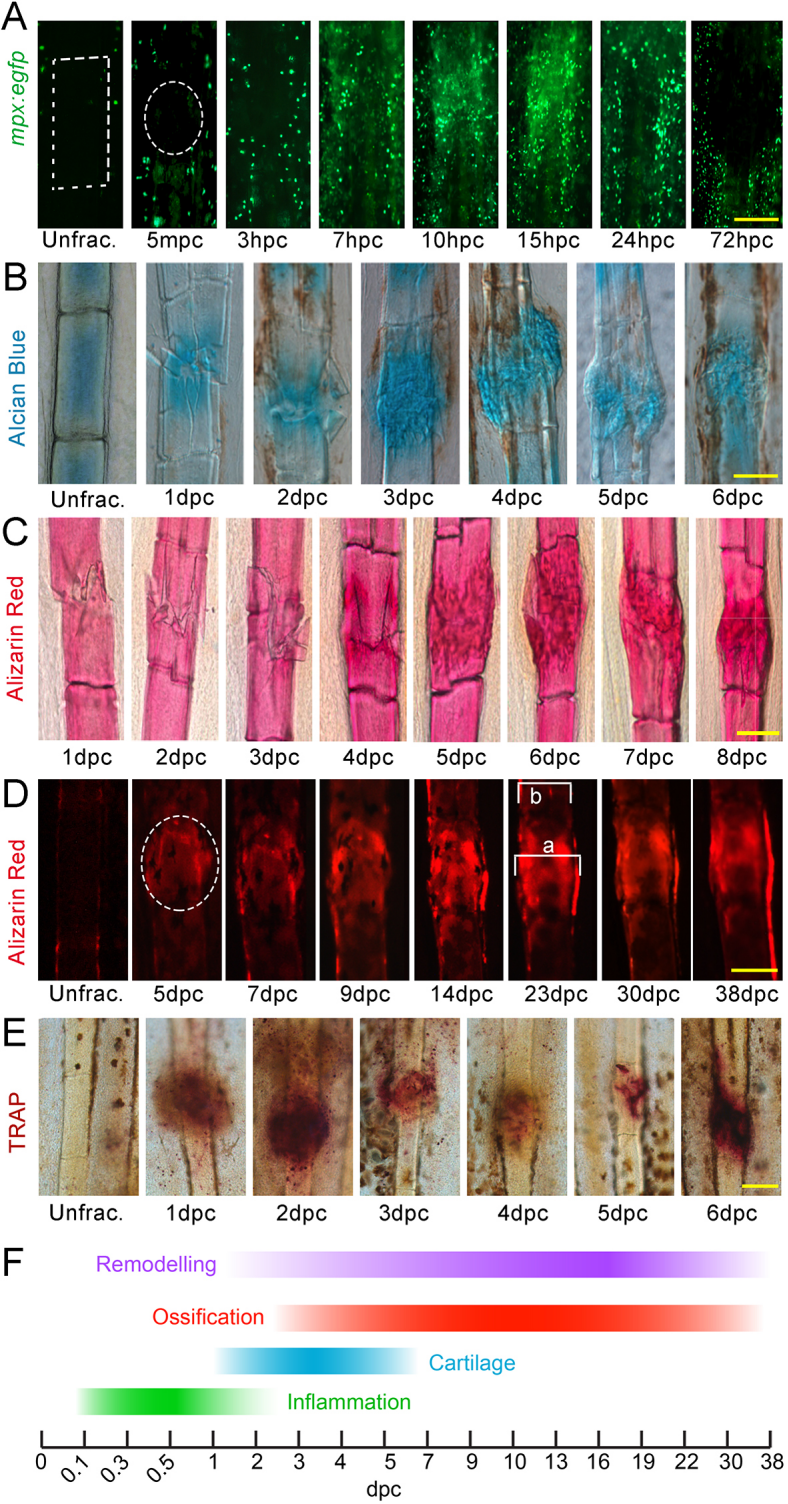
**Phases of bone crush fracture repair in the adult zebrafish fin.** (A-E) Fluorescent (A,D) and brightfield (B,C,E) images of *mpx:egfp* (A), Alcian Blue (B), Alizarin Red (C,D) and TRAP (E) labelling of lepidotrichia crush fractures at different stages of repair. Unfractured stainings are shown for reference. GFP-positive neutrophils (A), soft callus (B), hard callus (C,D) and osteoclast remodelling (E) have distinct temporal properties during repair. Fluorescent imaging of the bone callus (D) demonstrates the presence of a bone collar surrounding the callus. Relative callus width was measured by dividing the width of callus by the width of adjacent lepidotrichia (*a*/*b*) (D). Crush positions in A and D are circled; ray segment is outlined by a box in A. (F) Scheme showing the stages of repair. Scale bars: 100 µm. mpc, minutes post crush.

A large number of neutrophils, labelled by the *mpx:egfp* transgenic line, infiltrated the site between 3 and 7 h following crushing and peaked at 10-15 h post-crush (hpc). The inflammatory response slowly resolved following this and, strikingly, at 72 hpc neutrophils were absolutely excluded from all fracture sites to create a halo surrounding the area of repair (Fig. 1A). We also noted staining for Alcian Blue around the fracture from 1 day post crush (dpc), which peaked at 3 dpc (Fig. 1B). Using either chromogenic (Fig. 1C) or fluorescent (Fig. 1D) staining of bone by Alizarin Red, we then imaged the deposition of bone at the fracture site. A noticeable bone callus was formed around the fracture site from 5 dpc, forming on the existing cartilagenous template. In addition, a bone collar was evident along the edges of the ray when Alizarin Red staining was imaged fluorescently (Fig. 1D). The bone callus was thicker than the surrounding uninjured lepidotrichia and was evident until at least 38 dpc, but appeared to reduce in thickness slowly over time. As lepidotrichia can vary in thickness between fish, we measured the callus width as a proportion of the adjacent unfractured segment of the same ray to generate a measurement of relative callus width (e.g. length *a*/*b* in Fig. 1D). Over time, the callus could be seen increasing in relative width from 5 to 6 dpc, but then slowly resolved in thickness towards the original width (ratio *a*/*b*=1.0) by 38 dpc (Fig. S1). To assess whether thinning of the hard bone callus was a result of osteoclast-mediated repair, we visualised osteoclast activity using tartrate-resistant acid phosphatase (TRAP) staining. We observed persistent TRAP staining at all stages from 1 dpc, indicating long-term remodelling of the fracture and callus (Fig. 1E). Thus, the phases of repair are similar to those in mammalian fracture repair (Fig. 1F), yet show some temporal and spatial distinctions.

### OI model shows defective fracture repair

**Fig. 2.**
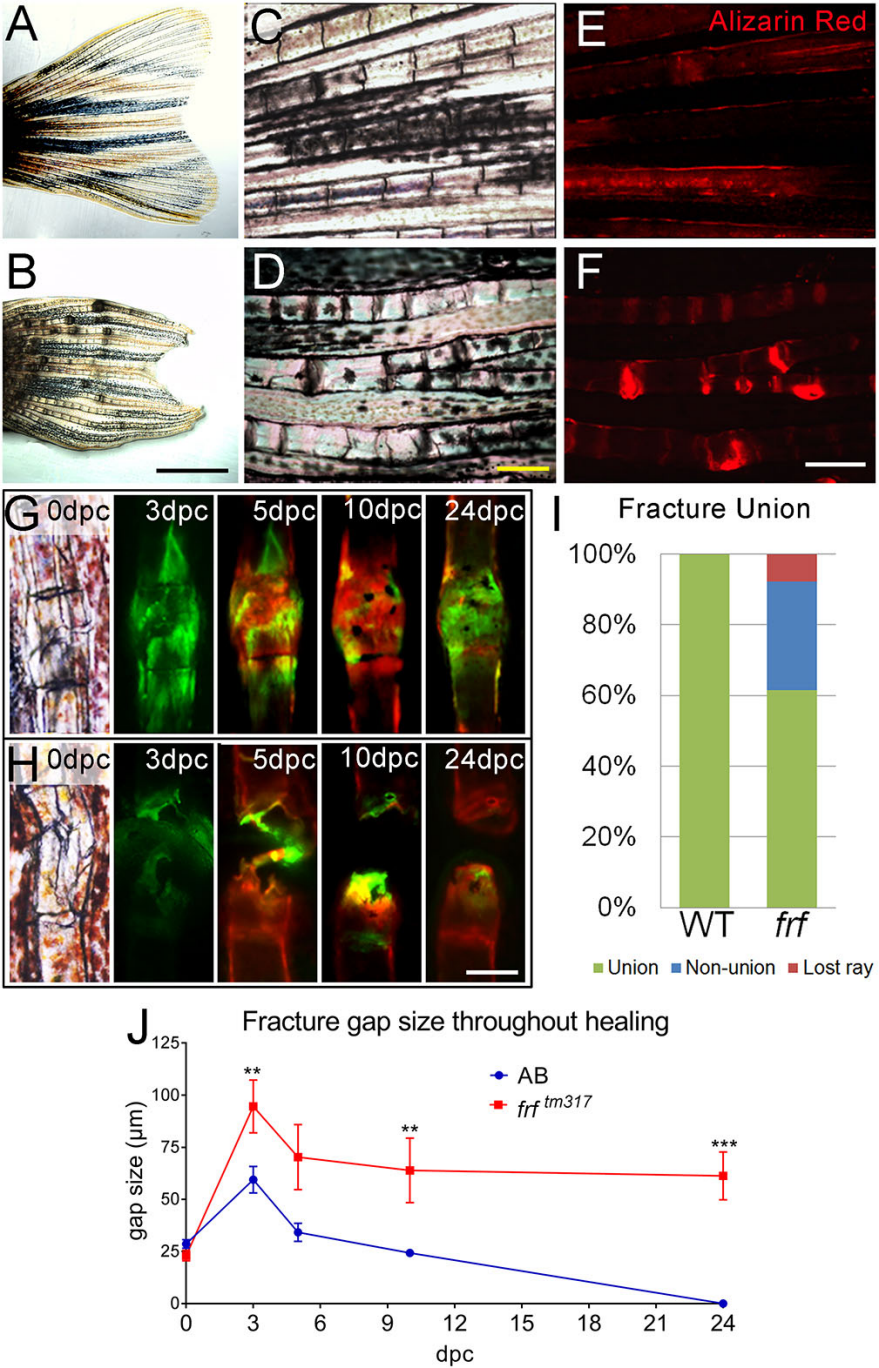
**Non-union fractures in a zebrafish OI model.** (A-D) Brightfield images at low (A,B) and high (C,D) magnification of adult fins at 3 months post fertilisation, showing thickening and dark foci in *frf* mutants (B,D) compared with WT (A,C). (E,F) Magnified images of WT (E) and *frf* (F) foci stained with Alizarin Red demonstrates bone calluses at these sites, indicating fractures. (G,H) Brightfield and fluorescent images of induced fractures in WT (G) and *frf* (H) at the indicated dpc. Bone was alternately stained with calcein (green) or Alizarin Red (red) to visualise callus growth. (I) A proportion of induced fractures in *frf* were non-union; *n*=12 per genotype. (J) The gap size following fracture was not reduced compared with WT over time; *n*=12 per point. **P<0.01, ***P<0.001; ANOVA with Sidak post test. Scale bars: in B, 2 mm for A,B; in D and F, 200 µm for C-F; in H, 100 µm for G,H.

We used our crush model to compare how fractures repair under genetic, pharmacological or bacterial stress. We first analysed a zebrafish model of OI, caused by mutation of *bmp1a* (Asharani et al., 2012). As noted previously, adult *bmp1a* mutants (*frf* mutants) have reduced bone densities and show spontaneous fractures in the lepidotrichia (Asharani et al., 2012) (Fig. 2A-F). These fractures showed strong associated Alizarin Red staining, in foci that were thicker than the adjacent fin ray, indicative of a bony callus and the repair process (Fig. 2F).

To determine whether OI zebrafish showed impaired fracture repair in addition to increased propensity for fracture, we tracked the healing phases of induced fractures. We fractured lepidotrichia of 3-month-old *frf* and WT adults and performed alternate staining of bone using calcein and Alizarin Red to track bone deposition over time. We initially noted a proportion of fractures that failed to heal and remained separated, even after 24 dpc (Fig. 2G,H), and termed these non-union fractures. These never occurred in WT fractures, but were seen in approximately 30% of OI zebrafish fractures; a further 8% of rays were lost, distal to the fracture site (Fig. 2I). Such non-union fractures are also seen in OI patients at similar rates (Gamble et al., 1988). To determine whether there was a defect at a specific time, we measured the gap between the rays for all fractures that failed to join. The gap was always larger in *frf* mutants than for fractures in WT fish, even at 3 dpc, indicating that there is an early defect during callus formation (Fig. 2J).

**Fig. 3.**
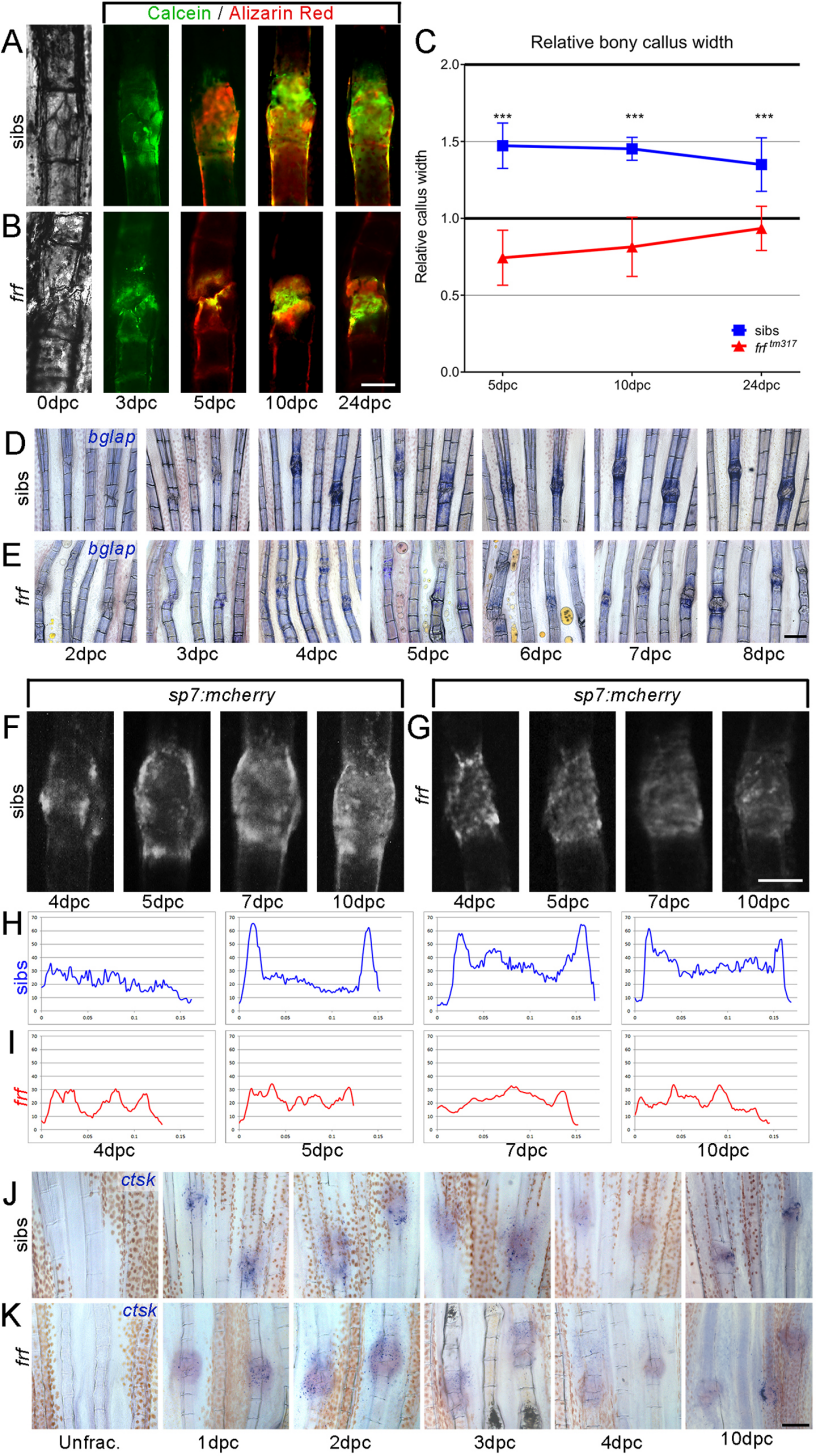
**Abnormal bone callus architecture in zebrafish OI fractures.** (A,B) Brightfield and fluorescent images of induced fractures in WT siblings (A) and *frf* (B) (at the dpc indicated) that underwent unification. Bone was alternately stained with calcein (green) or Alizarin Red (red) to visualise callus growth. (C) Bone callus formation in *frf* was delayed in comparison with WT, with relative callus width much lower at all stages measured (*n*=16 per time point). (D-G) Labelling of osteoblasts in induced fractures of *frf* (E,G) and siblings (D,F) at the indicated dpc, using *in situ* hybridisation for *bglap* (*osteocalcin*) (D,E) and confocal imaging of *sp7:mcherry* (F,G). No overt change in osteoblast recruitment was observed. (H,I) Intensity plots of mCherry fluorescence across the callus of sample images of WT (H) and *frf* (I) fractures, as measured from F,G. Osteoblasts did not form a marked collar around the callus and were more homogeneous across the callus in *frf*. (J,K) *ctsk in situ* labelling of osteoclasts in *frf* (K) and sibling (J) fin rays unfractured or following crush. Osteoclasts were present in both siblings and mutants following crush injury. ***P<0.001; ANOVA with Tukey post test. Scale bars: in B and G, 100 µm for A,B,F,G; in E and K, 200 µm for D,E,J,K.

We then analysed the fractures that joined and looked at callus formation using the same staining regime. A fracture callus in WT fish was almost 1.5 times the width of the ray by 5 dpc and then slowly reduced in width towards the original thickness, such that by 24 dpc it had a relative thickness of 1.35 (Fig. 3A,C). OI zebrafish fin rays formed calluses with significantly smaller relative width at 5 dpc, indicating a defect in deposition of bone to the callus. Where fractures repaired, the delayed deposition meant that the callus remained in the growth phase, slowly widening such that by 24 dpc they had almost grown to the original ray width (Fig. 3B,C).

We assayed the phases of induced fracture repair outlined above to determine which were altered in the OI zebrafish model. *In situ* hybridisation for the inflammatory cytokine *il1b*, which is evident in the fracture of WT fins at 8 hpc and 12 hpc, was also strongly expressed at those time points following fracture in *frf* fin rays (Fig. S2A,B). Similarly, after crossing the *mpx:egfp* transgenic line to *frf* and fracturing the fin rays, we noted no overt reduction in neutrophil recruitment, nor in the clearance of neutrophils from the fracture observed at later stages (Fig. S2C,D). We quantified the extent of neutrophil eGFP signal at the fracture site by measuring total fluorescence in the fracture area (corrected for background) at six time points between fracturing and 86 hpc. No significant difference was noted at any time point (Fig. S2E).

To assess whether a soft callus defect preceded the bone callus defect in OI fractures, we stained fractures with Alcian Blue and measured the relative soft callus width, but both WT and *frf* crushes contained equivalent Alcian Blue staining at the site (Fig. S3A,B). The relative width of the Alcian Blue stain at the crush site suggested a mild reduction in width at later stages in *frf*, but this did not reach significance at any time point (Fig. S3C). Thus, the bone callus defects are not presaged by deficiency in earlier repair phases.

This led us to use osteoblast markers to determine whether there was a change in osteoblast marker expression or distribution at the callus that might account for the altered bone callus in OI fish. *In situ* hybridisation staining indicated that fractures expressed higher levels of *bone gla protein* (*bglap* or *osteocalcin*) than adjacent unfractured ray segments by 4-5 dpc in both WT and *frf* (Fig. 3D,E); however, there was some variability. The *sp7:mcherry* (*osx:mcherry*) transgenic line (Spoorendonk et al., 2008) showed a stark increase in mCherry fluorescence at the fracture site, above basal levels outside the fracture site. Such osteoblast marker dynamics have been reported previously in fin ray fractures, where there is de-differentiation, localised migration and re-differentiation of osteoblasts at fracture sites (Geurtzen et al., 2014). We plotted mCherry fluorescence across the fracture in the WT over time through intensity mapping. Strikingly, at 5 dpc there was a rapid increase in fluorescence signal at the callus periphery, which was retained until at least 10 dpc, whereas the signal at the core of the callus gradually increased in intensity (Fig. 3F,H). In contrast, OI fractures showed a comparable increase in mCherry fluorescence in the callus core at 5 dpc, but the callus never displayed the peripheral fluorescence seen in the WT (Fig. 3G,I). The bone callus resolved after 5 dpc in WT fractures (Fig. 3C; Fig. S1), at a time when the callus was still growing in width in OI fish fractures. We then used *in situ* hybridisation for *cathepsin k* (*ctsk*) to see whether there was a change in the recruitment of osteoclasts to the fracture; however, staining was present at fractures in both cases at all time points assessed up to 10 dpc and we concluded that there was no change in osteoclast recruitment to the fracture (Fig. 3J,K). Thus, all cells of all phases appear at the fracture site in *frf*, yet the hard callus presents as dysmorphic, indicative of structural deficiencies in the osteoid produced.

### Bisphosphonates alter fracture callus dynamics

Bisphosphonates reduce osteoclast-mediated bone resorption, thus increasing bone density, and are first-line treatments for certain bone pathologies, including OI. Bisphosphonates are also reported to alter fracture callus formation and remodelling in several animal models, with different outcomes depending on the treatment regime and concentration (Fu et al., 2013; Hao et al., 2015; McDonald et al., 2008; Savaridas et al., 2013). We wanted to determine the parameters of alendronate treatment tolerated by zebrafish for future analysis of fracture repair. WT fish were treated by immersion in alendronate using a range of concentrations (0-500 μg/ml) for 24 h, both before and following fracture (−1 dpc to 1 dpc; 48 h treatment). Calluses were stained with calcein. Although a bone callus was still apparent at 4 dpc for lower concentrations, ray segments distal to the fracture site were often lost upon exposure to 250 and 500 μg/ml concentrations (Fig. S4A-C). In addition, in some cases, the immediate region of the fracture was lost and it appeared as a non-union fracture with non-healing skin wounds. Intriguingly, where rays were lost, regeneration was blocked, even 20 days after removal from the drug (Fig. S4B).

**Fig. 4.**
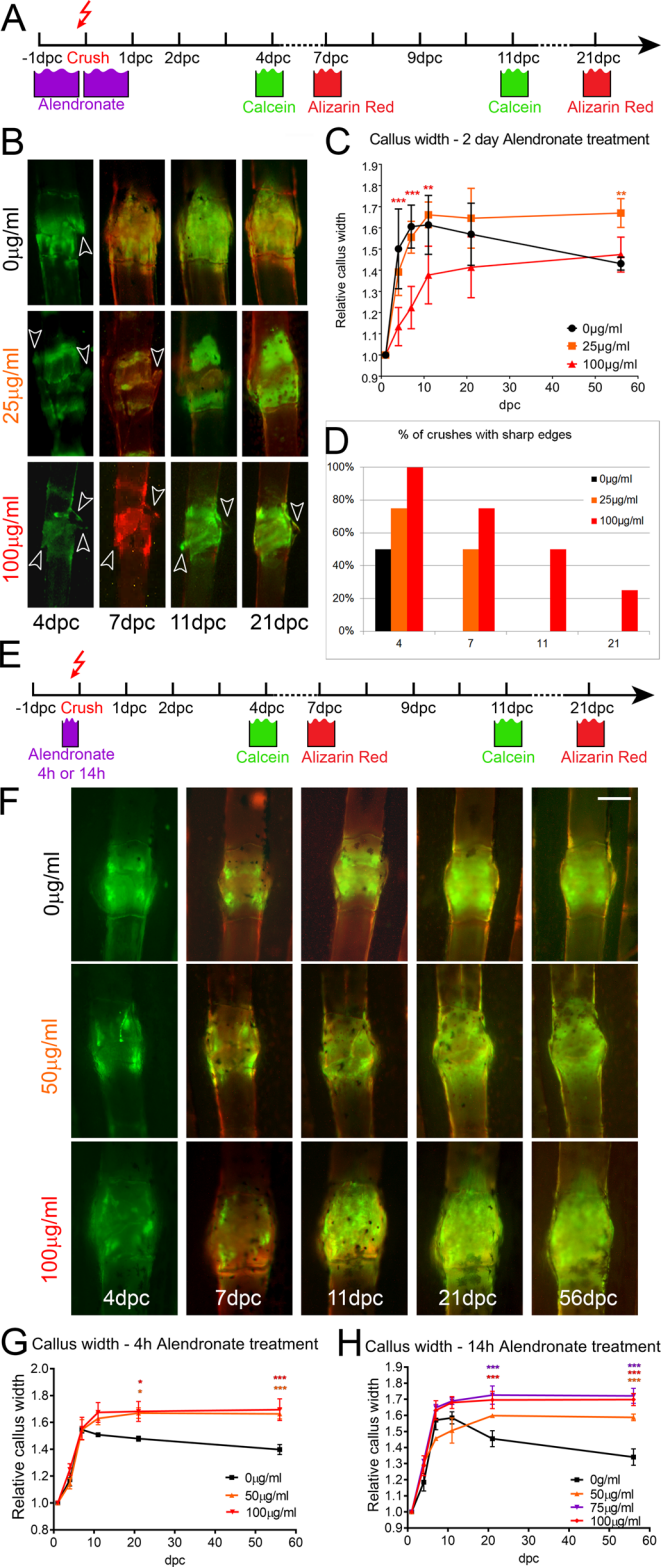
**Bisphosphonate treatment alters bone callus resorption dynamics.** Effect of extended and pulse exposure to alendronate on fracture bone callus. (A,E) Outlines of experimental regimes for extended (A) and pulse (E) treatment of fractures with alendronate and subsequent imaging. Fish were either treated for 1 day either side of the fracturing (A) or for a 4 h or 14 h pulse prior to fracturing (E). (B,F) Fluorescent images of fractures treated with indicated dose of alendronate following extended (B) or pulse (F) exposure as per regimes in A and E, respectively. The time points following crush are given. Arrowheads indicate bone debris. (D) Percentage of crushes showing bone debris (*n*=8 per point). (C,G,H) Relative callus width measurements at different time points for fractures treated with 25, 50, 75 or 100 µg/ml alendronate compared with untreated fractures for extended exposure (C), 4 h pulse pretreatment (G) or 14 h pulse pretreatment (H). Both alendronate concentration and time affected callus formation or resorption (*n*=4 per point). **P<0.01, ***P<0.001; ANOVA with Tukey post test. Scale bar: in F, 100 µm for B,F.

We thus proceeded to test 48 h bisphosphonate treatment at concentrations below 100 μg/ml and assess the effect on callus appearance and size. Tracking callus formation over time by alternating calcein and Alizain Red staining (Fig. 4A) and measuring the relative callus width demonstrated that, at 25 μg/ml alendronate, bone was deposited at a similar rate as in control fractures over the first 11 dpc (Fig. 4B,C). Exposure to 100 μg/ml of alendronate for 24 h before and after fracture, however, led to a smaller bone callus width at 11 dpc (Fig. 4C). Interestingly, the relative width of control calluses began to reduce after 11 dpc; however, this resolution was not apparent in fractures treated with either dose of alendronate as the callus widths had not reduced by 56 dpc (Fig. 4C). In untreated WT fish, bone debris, shards and sharp edges were apparent 4 days following fracture in control rays, and were largely cleared by 7 dpc. However, consistent with a loss of bone resorption, treatment with alendronate prolonged the appearance of debris and bone edges such that 25% of crushes exposed to 100 μg/ml retained sharp edges at 21 dpc (Fig. 4B,D).

As prolonged exposure to 100 μg/ml alendronate appeared to delay callus formation in addition to resolution, we next tested the effects of shorter exposure to alendronate, testing either a single 4 h or 14 h exposure immediately prior to forceps crush (Fig. 4E). We measured relative callus width following fracture for a range of concentrations from 50 to 100 μg/ml. We noted that for shorter pulse exposures, all concentrations of alendronate showed callus formation comparable with controls, yet measurably reduced remodelling as callus width did not decrease after 11 dpc and remained wide up to at least 56 dpc (Fig. 4F-H). Thus, short duration exposure to alendronate had identical effects to longer exposures. Both the inability to clear bone debris and the lack of callus remodelling suggested that osteoclast function was indeed reduced upon alendronate exposure.

**Fig. 5.**
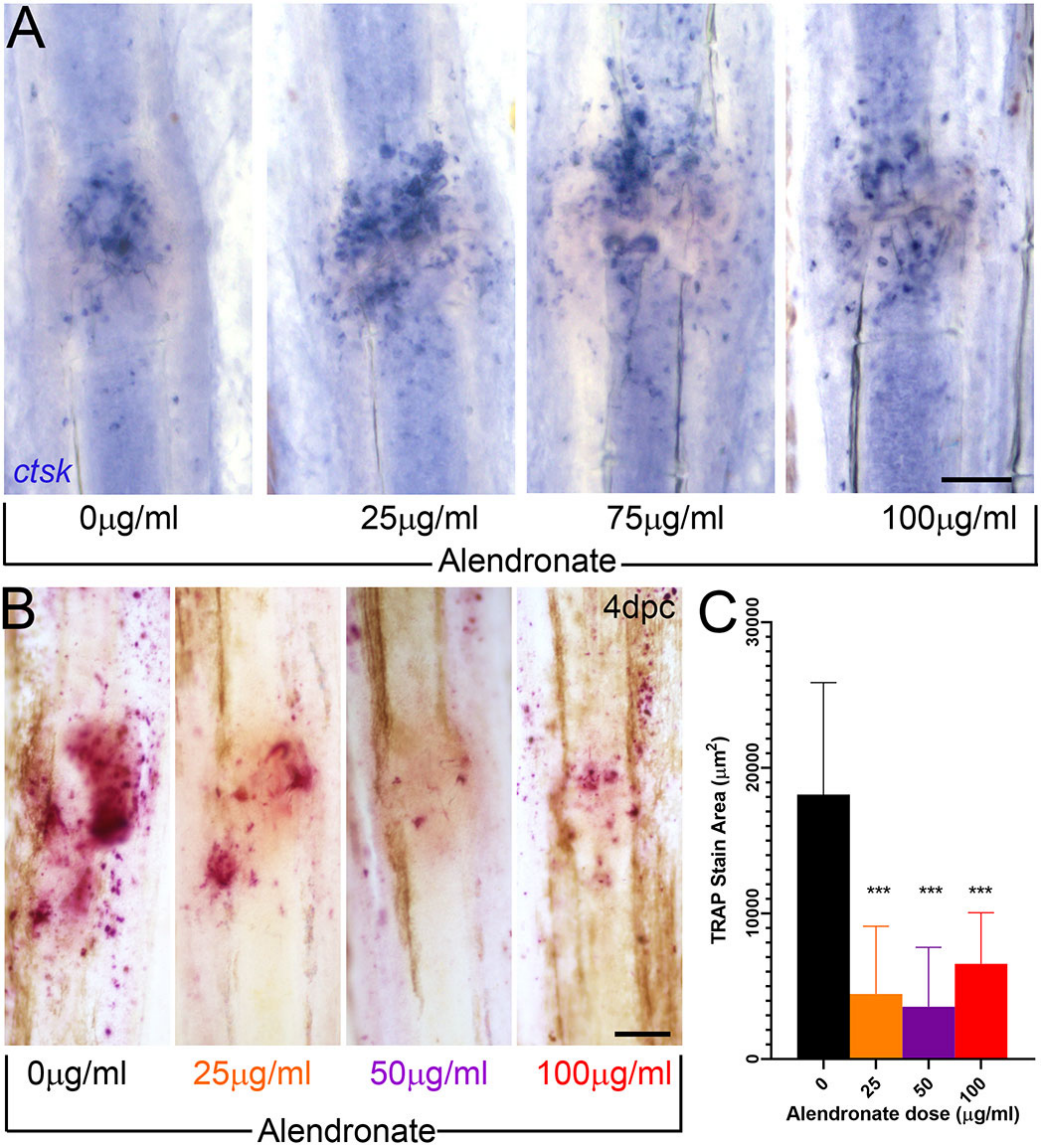
**Alendronate reduces osteoclast activity but not numbers at the fracture.** (A) Images of fractures processed by *in situ* hybridisation for *ctsk* to label osteoclasts at the fracture site at 1 dpc, following 14 h pulse exposure to various concentrations of alendronate, as shown. (B) TRAP staining performed at 4 dpc, following 14 h alendronate treatment at various concentrations immediately prior to fracture. (C) Reduced areas of TRAP staining were quantified (*n*=12). ***P<0.0001; ANOVA with Tukey post test. Scale bars: 100 µm.

We also examined osteoclast recruitment to fractures by *ctsk in situ* hybridisation following a 14 h pulse exposure to different concentrations of alendronate immediately prior to crush. At 1 dpc, osteoclasts were recruited at all concentrations, suggesting that cell number is not grossly affected by alendronate (Fig. 5A). We quantified osteoclast activity through measuring the extent of TRAP staining surrounding a fracture, following a 14 h exposure to a range of alendronate concentrations. We noted that at 4 dpc, all three concentrations showed significant reduction in TRAP staining (Fig. 5B,C). The effects of bisphosphonates in medaka also point to disruption of cell function over cell survival (Yu et al., 2016).

### Genetic ablation of osteoclasts reduces fracture callus remodelling

**Fig. 6.**
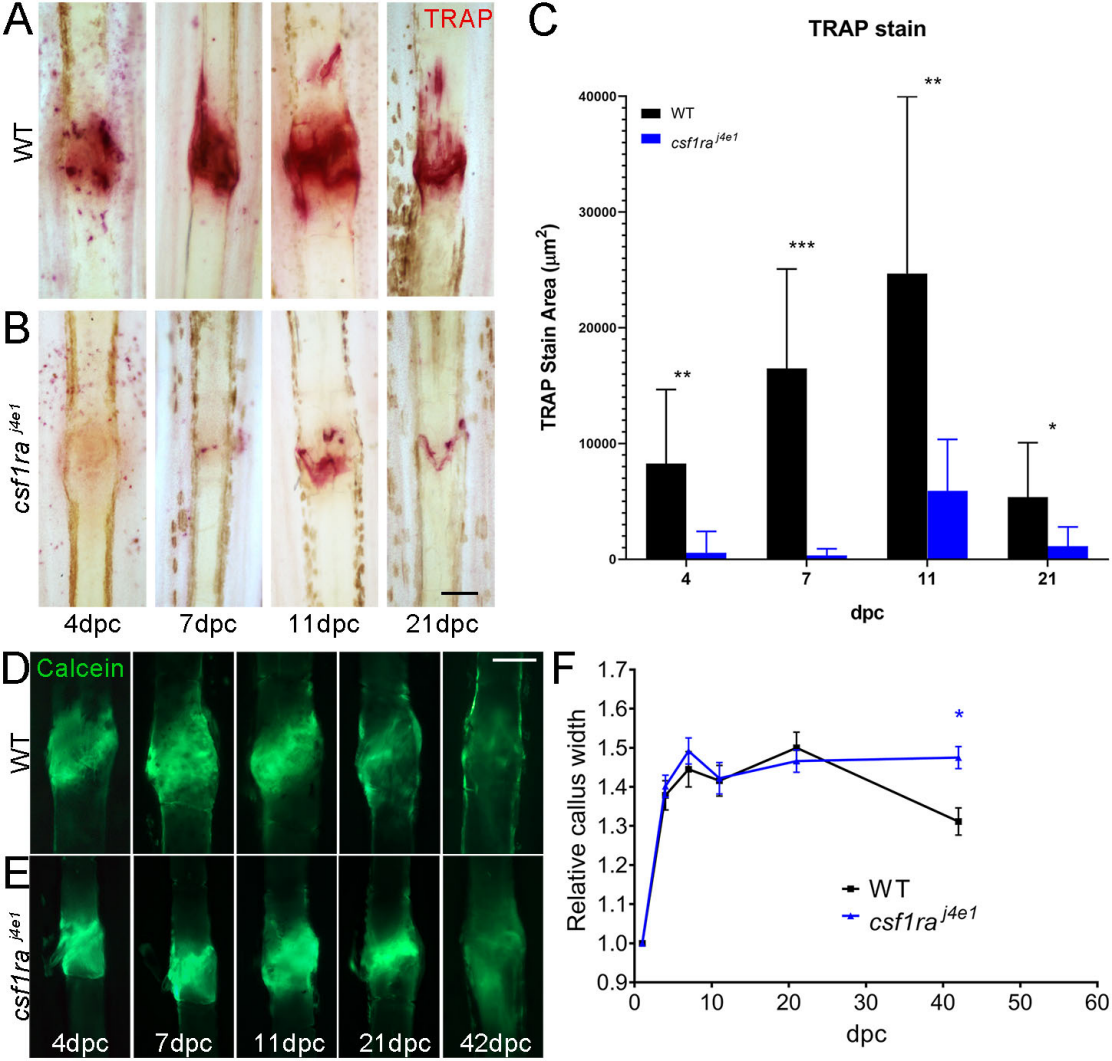
**Genetic ablation of osteoclasts reduces bone callus resorption.** (A,B) TRAP staining of fractures in WT (A) and *csf1ra^j4e1^* mutants (B) at given time points following crush. (C) The area of TRAP staining was significantly reduced in the mutants at all time points (*n*=12). (D,E) Fluorescent images of WT (D) and *csf1ra^j4e1^* mutant (E) fractures stained with calcein. The time points following crush are given. (F) Relative callus width measurements at different time points for fractures of WT and *csf1ra* mutants at given time points (*n*=15). By 42 hpc, the mutants had significantly fewer remodelled calluses. *P<0.05, **P<0.01, ***P<0.001; ANOVA with Sidak post test. Scale bars: in B and D, 100 µm for A,B,D,E.

To verify that the impaired callus remodelling induced by bisphosphonate treatment was a result of reduced osteoclast function, we used the *csf1ra^j4e1^* mutant, which has significantly reduced numbers of osteoclasts (Chatani et al., 2011; Parichy et al., 2000). We confirmed that these mutants have reduced osteoclast activity by TRAP staining at all time points following fracture (Fig. 6A-C). We then compared hard callus remodelling following fracture between WT and *csf1ra^j4e1^* mutants. Similar to bisphosphonate-treated fractures, these mutants showed reduced callus remodelling at 42 dpc (Fig. 6D-F), with sustained callus widths in the mutant.

### Bisphosphonates reduce spontaneous fractures in the OI zebrafish model

**Fig. 7.**
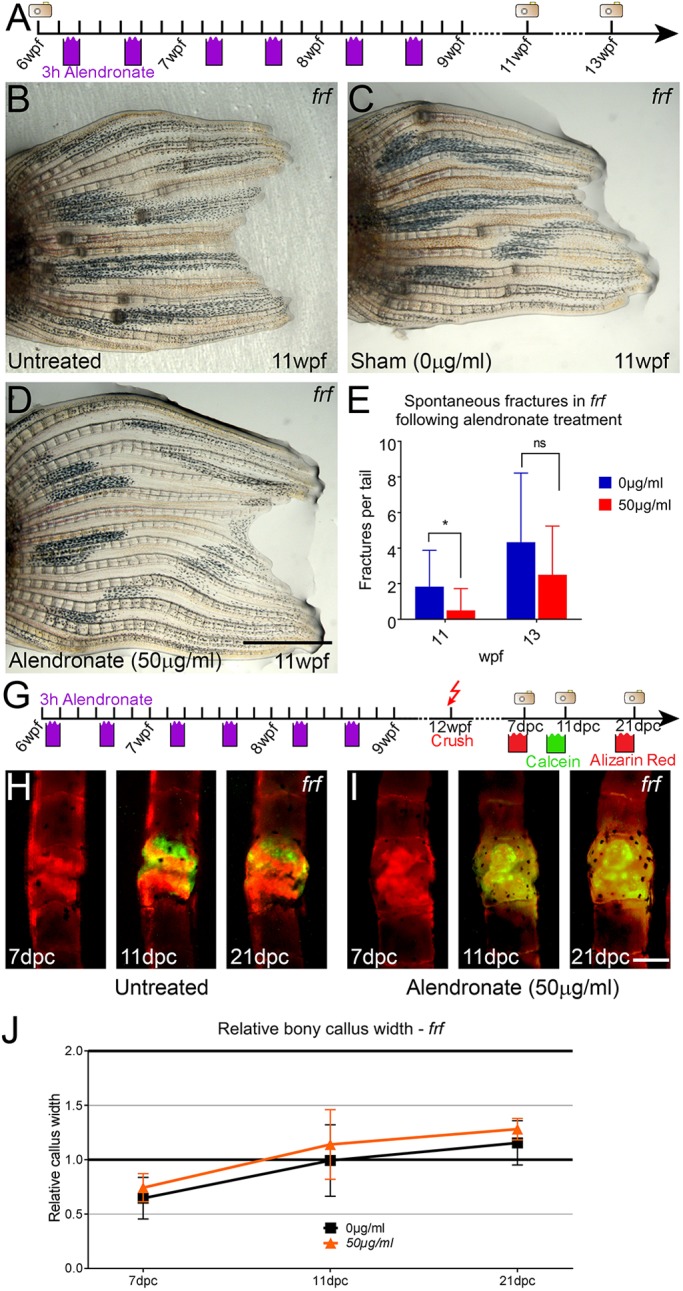
**Alendronate reduces fractures in the zebrafish OI model.** (A) Outline of experimental regime for cyclical treatment of *frf* with 50 µg/ml alendronate and subsequent imaging of fins for spontaneous fracture occurrence. (B-D) Brightfield images of tail fins of *frf* fish at 11 weeks post fertilisation (wpf), either untreated and directly imaged (B), cyclically immersed in 50 µg/ml alendronate twice a week (3 h each day) for 3 weeks (D) or sham treated, where fish were immersed in normal tank water to recapitulate handling of the OI fish (C). (E) Number of spontaneous fractures observed was counted at both 11 wpf and 13 wpf (*n*=7 per point; Mann–Whitney test). (G) Outline of experimental regime for cyclical treatment of *frf* with 50 µg/ml alendronate, fracturing and subsequent imaging of fins following alternate staining with calcein (green) or Alizarin Red (red) to visualise callus growth. (H,I) Fluorescent images of *frf* fractures either treated with 50 µg/ml alendronate (I) or untreated (H) and imaged for calcein or Alizarin Red at the indicated time points. (J) The relative bone callus width was compared, but no difference in callus growth was noted upon treatment with alendronate (*n*=8 per point). *P<0.05; ANOVA with Tukey post test. Scale bars: in D, 2 mm for B-D; in I, 100 µm for H,I.

Because bisphosphonates reduce osteoclast function in zebrafish fractures and are used clinically to reduce fracture occurrence in OI patients, we asked whether alendronate treatment could reduce the spontaneous fracture rate in *frf* mutants. We replicated the cyclical bisphosphonate treatment used for patients, giving 3 h pulse treatments of 0 µg/ml (sham) or 50 µg/ml alendronate twice a week for 3 weeks (total of six treatments). As there were no spontaneous fractures evident in *frf* at 6 weeks of age, but there were by 11 weeks of age, we initiated the treatment at 6 weeks, and imaged fins at 11 and 13 weeks (Fig. 7A). We observed a significant reduction in spontaneous fractures in treated *frf* fish at 11 weeks following exposure to alendronate compared with untreated sham fish, which were subjected to an equivalent regime of repeated handling over the 5 weeks (Fig. 7B-E). However, such a reduction was temporary and fractures reappeared in alendronate-treated fish by 13 weeks of age, 5 weeks after cessation of treatment (Fig. 7E).

We also asked whether cyclical treatment of *frf* bone with alendronate through the 3 week treatment regime outlined above could also improve the callus size during fracture repair. To answer this, we pretreated 6-week-old *frf* juveniles with 50 µg/ml alendronate as above and then induced a crush fracture at 13 weeks of age (Fig. 7G). We then monitored the dynamics of callus growth over the next 21 days, measuring relative callus width. As shown earlier, *frf* fracture calluses are initially dysmorphic and hypotrophic compared with WT calluses (Fig. 3A-C). Treatment of bone with alendronate did not appear to restore bone callus formation, which remained delayed (compare Fig. 7H-J with 3A-C). Furthermore, because the calluses remained in growth phase for longer, they did not enter into the remodelling phase and so there was no appreciable effect on treated and untreated *frf* fractures at any stage analysed. Our previous analysis of spontaneous fractures in *frf* juveniles suggested that the effect of alendronate is temporary. As the final callus measurement occurred 7 weeks after the last exposure to alendronate, we repeated the analysis on an induced fracture in *frf*, exposing the fish to alendronate for a 14 h pulse immediately prior to performing a crush fracture. There was no noticeable difference between the kinetics of callus growth or resolution in cyclically treated *frf* fractures and untreated *frf* fractures (Fig. S5A-D).

### Persistent *Staphylococcus aureus* infection inhibits fracture repair

**Fig. 8.**
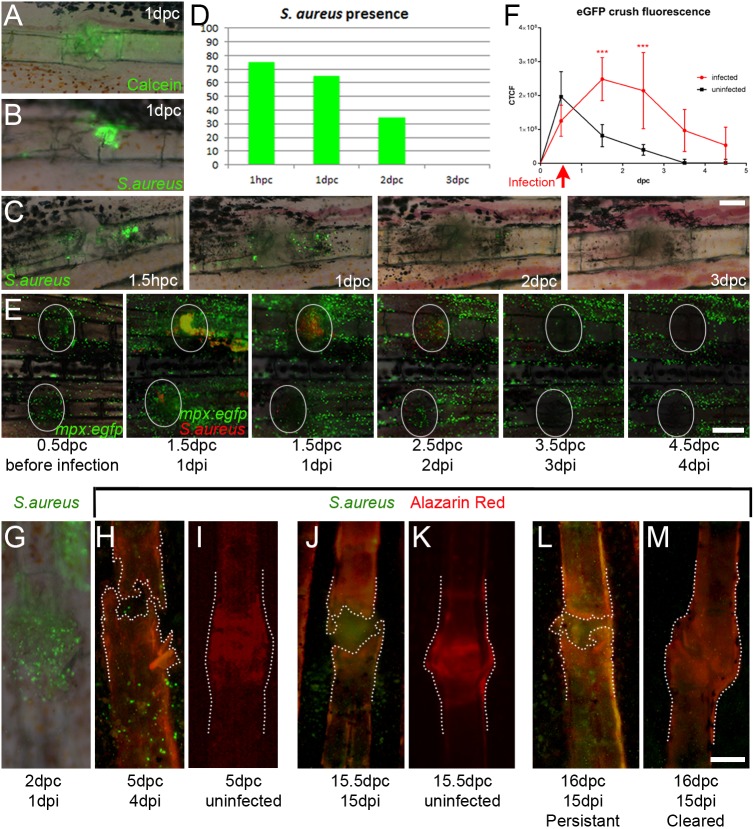
***S. aureus* infection of fractures delays healing.** (A-C) Fluorescent images overlying Nomarski images of either calcein tracer (A) or eGFP-labelled *S. aureus* (B,C), showing that both can be successfully introduced to a fracture site. (D) Injection of 0.5 nl of 2500 cfu/nl *S. aureus* yielded detectable bacteria up to 3 dpi (*n*=20 per point). (E) Fluorescent images of *mpx:egfp*-positive neutrophils (green) and mCherry-expressing *S. aureus* (red) over 4 days of infection introduced 12 h after crushing. (F) Inflammation correlates with the presence of *S. aureus* and prolongs inflammatory response at the fracture (*n*=4 per point). ***P<0.001; ANOVA with Sidak post test. (G-M) Fluorescent images of eGFP-expressing *S. aureus* introduced either 12 (J) or 24 h (G,H,L,M) following fracture and viewed with either Nomarski optics (G) or with fluorescence for Alizarin Red (H-M). (I,K) Corresponding uninfected fracture controls are shown. Outlines indicate bone stained by Alizarin Red, showing that the presence of *S. aureus* predicts poor callus formation compared with uninfected controls (I,K) or where the infection has been cleared (M). Scale bars: in C and G, 100 µm for A-C,G-M; E, 200 µm.

We have shown the utility of our fracture model for defining repair progression under clinically correlated conditions. As *S. aureus* infection is a major impediment to fracture healing in the clinic, and of increasing concern worldwide, we augmented the fracture model by developing a means to infect the fin ray following fracture. We first assessed the feasibility of introducing 0.5 nl of either an inert tracer fluid (0.005% calcein) or GFP-expressing *S. aureus* (at 2500 cfu/nl) following bone fracturing, using a glass injection needle and delivering into the crush site or proximal exposed intra-ray cavity. We were able to consistently observe calcein or bacteria in the region (Fig. 8A,B) immediately following injection. Although calcein dissipated after 2 days, we could observe bacteria up to 3 days post infection (dpi). A time course of bacterial presence showed that at 0.5 h post infection (hpi), (i.e. 1 hpc), 75% of injected crushes contained visible bacteria. The load of bacteria reduced over the next 3 days and was gone by 3 dpc (Fig. 8C,D). We attempted to prolong infection duration by increasing the infection load through either increasing the injection volume or *S. aureus* concentration. By injecting 4 nl of *S. aureus*, there was a high rate of loss of the fin ray distal to the injected fracture (72%). This occurred immediately (within 1 h) after injection, suggesting that it was due to mechanical disturbance from excess fluid, rather than bacteria. Such fin rays often had detectable bacteria at the injection site at least until 7 dpi, and fin rays never initiated regeneration until all bacteria were cleared from the site (Fig. S6A-C). Following clearance, all infected lepidotrichia showed impeded or abnormal regeneration up to at least 2 weeks post infection (Fig. S6D-G).

Where fin rays were not lost, 30% of fractures retained detectable *S. aureus* at 7 dpi (Fig. S6H); however, because of the high prevalence of fin ray loss, we introduced more bacteria to the crush by increasing the concentration (7500 cfu/nl) and returning to injections of 0.5 nl. We also delayed infection until 12 or 24 h after the fracture to allow the site to settle before challenging with infection. We often saw bacteria within the fracture, or displaced within the adjacent fin-ray segment, up to 4 dpi, suggesting that we could generate a more sustained infection with this concentration. By injecting mCherrry-labelled *S. aureus* into fractured fins of *mpx:egfp* adults, we could directly observe the inflammatory response, noting a robust infiltration of neutrophils from 1 dpi, well above the normal levels in an uninfected fracture. Levels of inflammation correlated strongly with bacterial load in the fracture, and neutrophils intermingled with bacteria in the fracture zone. We quantified the inflammatory response by measuring eGFP fluorescence as described above and observed a significant increase in inflammation at 1 and 2 dpi, but also a persistence of neutrophils (Fig. 8E,F).

To determine the effect of bacteria on bone repair, we stained infected fractures with Alizarin Red. At 1 dpi, eGFP-positive bacteria could be seen in the fracture (Fig. 8G) and persisted until 5 dpc, although many were slightly displaced from the site by then (Fig. 8H). Where bacteria could still be observed in the fractured ray, the bone remained unrepaired, with no bone callus or mineral deposit between the rays (Fig. 8H); by contrast, uninfected fractures had all formed a hard callus by 5 dpc (Fig. 8I). Fractures were also checked at 15 dpc. At this point, many of the infected fractures were now clear of bacteria and had a large callus, appearing similar to uninfected fractures at this stage (Fig. 8K,M). If bacteria were retained in fractures at 15 dpi, there was a more homogenous distribution and individual colonies were not readily seen. In these cases, there was an absence of visible callus and lack of bone joining (Fig. 8J,L). We conclude that if *S. aureus* can evade clearance, it can block normal fracture healing in zebrafish.

## DISCUSSION

We show that the zebrafish fin crush model is a rapid and simple means of tracking fracture repair at the tissue and cellular levels, allowing easy visualisation and imaging of fin rays at all stages and assisting interrogation of phases through the growing battery of markers and transgenic lines available for cells of the fish skeleton. The model is enhanced by the relative low cost of adult zebrafish compared with mammalian systems; furthermore, each fin has 18-20 rays per caudal fin (Parichy et al., 2009), allowing multiple equivalent fractures per individual; together these increase statistical power for fracture studies. As in humans, zebrafish have a reproducible phased response to fractures, which are similarly sensitive to a number of perturbations. We have shown that a genetic defect in mature collagen I production, excessive bisphosphonate exposure or bacterial infection lead to delayed or non-union repair in zebrafish.

We employed this model to track the repair process for a zebrafish model of OI, *frf*, caused by mutations in Bmp1a, the collagen I C-propeptidase. We demonstrated that the increased rate of non-union seen in OI patients is also modelled in *frf* and that the major defect appears to be a result of impaired formation of the bony callus, which appears hypoplastic and delayed. Because markers of osteoblasts appear at the fracture at comparable times in both WT and OI fractures, we propose that the OI callus defect is probably caused by lack of sufficient mature collagen I. Indeed, this defect mirrors the delayed development of bone in the larval stages of *frf* (Asharani et al., 2012). Although BMP1 is also described as the C-propeptidase for the collagen II of cartilage (Kessler et al., 1996), we did not see a significant reduction in soft callus size, as we did for the hard callus. Cartilage callus might be less disrupted by retention of collagen II C-propeptides, as the Bmp1 mouse and fish mutants present bone defects without cartilage defects. That a soft callus forms well, but does not progress to a hard callus, reflects what is known about the composition of patient non-unions, which are fibrocartilage in nature (Panteli et al., 2015).

We were also able to show that application of the bisphosphonate alendronate to the fish tank water was sufficient to reduce fracture rates in the *frf* model. However, this effect appeared transient as the effect was not significant 2 weeks later, although the trend remained. Extended or alternate treatment regimes could improve the efficacy of fracture suppression. Such a response to bisphosphonate is well described in other animal models of OI (Camacho et al., 2001). The ease of application by direct immersion and the numbers of individuals that can be treated make zebrafish an intriguing system for comparative or longitudinal drug trials, especially for assaying multiple regimes in parallel. We initially used high concentrations of bisphosphonate in our analyses, but these showed an inhibitory effect on repair. Indeed, high doses led to loss of all rays distal to the fracture site and, intriguingly, a complete failure to regenerate, even 21 days following crushing. In some cases, the fracture was never repaired and there was a concomitant failure to repair the overlying epidermis. Thus, high levels of bisphosphonate can block regeneration and repair for an extended period. Such an outcome is not apparent for the low doses used clinically, where alendronate is considered to be safe; however, some rat studies have shown that higher doses of alendronate are deleterious to both osteoclasts and osteoblasts (Sama et al., 2004). One major concern of anti-resorptives is osteonecrosis of the jaw, the underlying mechanism of which has not been fully determined (Wat, 2016). It might be that there are commonalities between bisphosphonate-induced jaw necrosis and the block in repair of fin ray fracture and regeneration seen at high doses.

Although the *frf* OI zebrafish model is a result of mutations in the collagen I C-propeptidase Bmp1a, all other described zebrafish OI models are caused by mutations in collagen I genes (Asharani et al., 2012; Fisher et al., 2003; Gistelinck et al., 2018; Henke et al., 2017), including both dominant and recessive mutations and representing a series of phenotypic severity. It would be useful to assess whether spontaneous and non-union fractures occur in these models as well, and to attempt to alleviate the fracture rate with bisphosphonates. Elegant work has recently shown that a chemical chaperone can ameliorate the bone defects of the *col1a1a* zebrafish mutant *chihuahua* (Gioia et al., 2017) and the cell stress of OI patient cells (Besio et al., 2018). This, combined with our demonstration of the efficacy of alendronate in *frf*, promote zebrafish OI models as being relevant for testing drug regimes.

Although the fracture repair phases are globally similar to those in humans, there are some zebrafish-specific temporal differences. For example, TRAP staining, indicative of osteoclast activity, is apparent much earlier in the repair process in zebrafish, possibly to clear debris rapidly. Both genetic (*csf1ra* mutants) and pharmacological (alendronate) disruption of osteoclasts indicate that they are crucial for later remodelling and removal of the hard callus, as seen in human fractures. This zebrafish callus resolution model presents a quantifiable assay for osteoclast activity in fractures. One of the most striking observations from the temporal and spatial analysis of cellular constituents within the fracture was the behaviour of neutrophils. Although they are recruited very rapidly after fracture, and persist for 3 days, they are then excluded completely from the fracture site. There is a similar and crucial switch from a pro-inflammatory to an anti-inflammatory fracture environment in humans, and delaying this switch is thought to inhibit repair through disruption of angiogenesis (Schmidt-Bleek et al., 2015). The dynamics of neutrophils in mammalian bone fractures is poorly defined. From limited studies it is clear that neutrophil numbers are regulated at the fracture at different times, and that induction of systemic inflammation can abort fracture repair, although neutrophils do not seem to be the cell type directly impeding repair in this model (Kovtun et al., 2016). However, reduction in the number of neutrophils is also detrimental to fracture healing, indicating that the timing of neutrophil presence at the fracture is crucial in mice (Kovtun et al., 2016). There is far more information on the importance of T cells and macrophages on the fracture repair process and reports indicate that immunosuppression can lead to non-union, further highlighting the initiating role of inflammation in bone repair (Ono and Takayanagi, 2017). The dynamics of T cells, macrophages and B cells in zebrafish fracture is an important dataset to obtain in the future and to compare with mammalian studies. Furthermore, it would be of interest to determine the molecular signals dictating both the initial recruitment and subsequent exclusion of neutrophils to/from the site of repair. Such defined spatial and temporal exclusion suggests that neutrophils have an effect on bone repair, requiring their exclusion, although this currently remains speculation. In line with this, our data show the retention of neutrophils in *S. aureus*-infected fractures and the correlated repair block. There is a wealth of literature indicating the direct inhibitory effect of *S. aureus* on osteoblasts, although either insufficient or excessive inflammation is also known to be detrimental (Schmidt-Bleek et al., 2015).

We have used bacterial injection into the fracture site to model the clinical fracture complications of infection and prolonged inflammation, showing that bacteria and/or prolonged inflammation are detrimental to fracture repair. Although *S. aureus* is responsible for the vast majority of bone infections clinically, other flora have been implicated in osteomyelitis, including *Candida*, *Mycobacterium*, *Aspergillus* and *Pseudomonas* (Gomes et al., 2013)*.* It would be interesting to see whether our crush infection model can be used to demonstrate the ability of other relevant bacteria to colonise fractures and inhibit repair. In addition, as with alendronate treatment, we often noted that there was loss of distal rays with high bacterial doses and often failure to regenerate those while bacteria remained at the site. The effect of commensal and pathogenic bacteria on the regenerative capacity of tissues is an understudied field. Zebrafish appear well poised to address such issues of bacterial function in repair of tissues such as bone, a growing clinical issue.

## MATERIALS AND METHODS

### Zebrafish husbandry and lines

Fish were kept in the IMCB zebrafish facility and all methods performed under IACUC number #140924. Embryos were obtained through natural crosses, with staging as described by Kimmel et al. (1995). Homozygous *frilly fins^tp34^* or *frilly fins^tm317^* adult fins were used for analyses of OI fractures (Asharani et al., 2012), and both showed comparable defects. The *csf1ra^j4e1^* allele (Parichy et al., 2000) was obtained from R. Richardson, University of Bristol. The transgenic lines *Tg(mpx:GFP)^i114^* (Renshaw et al., 2006) and *Tg1(Ola.Sp7:mCherry)^zf131^* (Spoorendonk et al., 2008) were used to label neutrophils and osteoblasts, respectively.

### Lepidotrichia crush injury

Adult zebrafish, at 3 months post fertilisation, were anaesthetized in 0.013% Tricaine (buffered to pH 7.0) in facility water and placed on a petri dish under a Leica MZ7.5 dissecting stereomicroscope. Excess water was removed by paper tissue, the fish was positioned on its side and the tail was spread out evenly on the surface. Drummond number 5 forceps were used to perform a bone crush of lepidotrichia (Movie 1). Four crushes per tail were introduced, on the second and fourth ray from both dorsal and ventral sides of the tail at the second segment anterior to the first level of bifurcation. Bone crush was performed in the middle of the segment, with the minimal strength needed to crush through both of the hemirays without impacting adjacent tissues. After surgery, fish were returned to a tank filled with standard fish tank water for recovery.

### Tail collection and storage

Following crush fracture, tails were collected at the required repair stage by anaesthetisation in buffered Tricaine and dissection of the whole tail fin with a scalpel. Collected tails were placed in 4% paraformaldehyde (PFA) solution and kept at 4°C, with shaking, overnight. The following day, specimens were washed in PBST briefly, then moved through a methanol series consisting of 30, 60 and 100% methanol. Specimens were kept for 10 min in each solution and then stored in 100% methanol at −20°C.

### *In situ* hybridization

Whole-mount *in situ* hybridization was performed on dissected tail fins using the protocol adapted from Thisse and Thisse (2008). DIG-labelled RNA probes for *bglap*, *ctsk,* and *il1b* were synthesised from DNA templates that were PCR amplified directly from cDNA. The reverse primer contained a T7 sequence to allow RNA transcription using a T7 RNA transcription kit from Roche. Samples were cleared in glycerol before mounting for imaging.

### Alcian Blue staining

Tissues kept in 100% methanol at −20°C were rehydrated through a methanol series at room temperature (100, 60 and 30% methanol), washed twice with PBST for 10 min, then moved to Alcian Blue stain solution (1% HCl, 0.1% Alcian Blue in H_2_O) for overnight staining. Next day, the samples were washed for 15 min in PBST and bleached in bleach solution (0.3% H_2_O_2_, 1% KOH in water) for 30 min at 37°C, then washed in PBST and stored in glycerol.

### Alizarin Red bone staining

Specimens stored in 100% methanol at −20°C were moved through a methanol/PBS wash series consisting of 10 min washes in 100, 60 and 30% methanol, followed by two 10 min PBST washes. Subsequently, samples were pre-incubated in 0.5% KOH for 15 min and moved to 0.2% Alizarin Red solution in 0.5% KOH for overnight staining at room temperature. Next day, three sets of 5 min washes with 0.5% KOH were performed followed by 1 h hydrogen peroxide bleaching (3% H_2_0_2_ in 1% KOH). Upon completion, three sets of 10 min PBST washes were performed and samples were moved to glycerol for storage and imaging.

### Fluorescent bone staining *in vivo*

Alizarin Red S (Sigma-Aldrich, A5533) and calcein (Sigma-Aldrich, 17783) were used for fluorescent *in vivo* bone staining on live fish. Alizarin Red (0.001%) or calcein (0.0005%) in fish tank water was used for overnight staining. Fish were immersed in small tanks in 200 ml of staining solution the night before imaging. The following day, they were moved to clean water for a quick wash, anesthetized and imaged under a Zeiss fluorescent microscope.

### TRAP staining

To stain for osteoclast activity, we used the Acid Phosphatase Kit from Sigma-Aldrich Diagnostics. Adult zebrafish tail fins were left in the fixing solution (24% citrate solution containing 65% acetone and 8% formaldehyde) for 40 min at room temperature. The samples were removed from this solution by washing with PBST (3×5 min), and then stained according to the kit instructions. Tail fins were kept in the stain for 3 h in the dark, rinsed with PBST (3×5 min), post-fixed in 4% PFA for 30 min and stored in 75% glycerol. The samples were visualized by light microscopy.

### Bisphosphonate zebrafish treatment

Alendronate sodium trihydrate (Sigma-Aldrich, A4978) was dissolved in fish medium at concentrations of 25-500 µg/ml. Fish were treated by immersion in the drug solution for the indicated treatment times. For recovery, fish were returned to a tank of fresh water. Treated fish were kept in separate tanks, under standard husbandry conditions, with feeding and water changes performed as normal.

### *Staphylococcus aureus* injections

*Staphylococcus aureus SH1000*-derived strains expressing mCherry or eGFP (Prajsnar et al., 2008) were injected into the fracture site after the crush using glass needles pulled with a Sutter Instruments P-97 Flaming/Brown Micropipette Puller. The needle was inserted through the fracture inside the ray into the intra-ray space proximal to the breakage. Bacteria were injected while retrieving the needle from the fracture, introducing 0.5 nl or 4 nl of *S. aureus* at either 2500 cfu/nl or 7500 cfu/nl. After infection, fish were returned to separate tanks filled with standard tank water. Infected fish were kept separately in a designated rack. They were fed normally and water in the tanks was changed daily to avoid bacterial outbreak in the water. Calcein (0.005%) was injected as a control.

### Imaging and statistics

Samples were imaged on an Axio Imager Z2 (Zeiss), Zeiss LSM 700 confocal or Leica MZ16F stereoscope and processed using ImageJ. Callus sizes were measured using AxioVision software. The width of the callus was measured in micrometres and normalized against the width of an unbroken part of a proximal segment to omit ray size differences between individuals and obtain actual callus sizes. Data on callus size are presented and plotted on the chart as a proportion of unbroken bone. Corrected total crush fluorescence was used to measure fluorescent signal emitted by neutrophils in the fracture area of *Tg(mpx:GFP)* fish. The protocol for corrected total cell fluorescence was adapted from the literature (McCloy et al., 2014). Fluorescent channels were separated using ImageJ and the fracture area was defined using the green channel. The same fracture area was used for all time points, assuring consistency in measurements. All measurements were normalised against initial fluorescence detected at the fracture site just before an injury (mean fluorescence at 0 hpc). Statistics were performed using Prism software from GraphPad, using ANOVA with the Tukey or Sidak post-test for multiple comparisons or Mann–Whitney for pairwise comparisons.

## Supplementary Material

Supplementary information
