# Supplementary material for: Clinical pathologies of bone fracture modelled in zebrafish
Source: Dis Model Mech. 2019 Sep 3;12(9):dmm037630. doi: 10.1242/dmm.037630 (PMC6765199; doi:10.1242/dmm.037630)
Supplement: Supplementary information [file dmm-12-037630-s1.pdf]

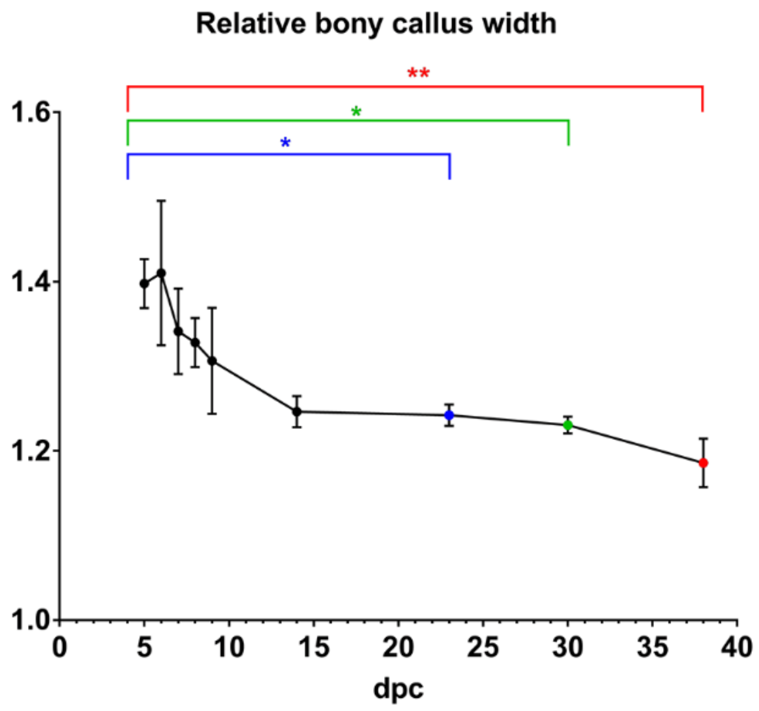

Figure S1: Change in Relative bony callus width during fracture repair
Ratio of width of callus to adjacent unfractured bone ray measured over days post crush (dpc). Ratio is measured as shown in Figure 1D (a/b). The callus initially forms to be 1.4 times wider than adjacent ray and is slowly remodelled over 38 days. n=4 per ANOVA point. ANOVA with Sidak post test.; * = $p<0.05$; ** $p<0.01$. Scale Bars B,D=100µm.

Disease Models & Mechanisms • Supplementary information

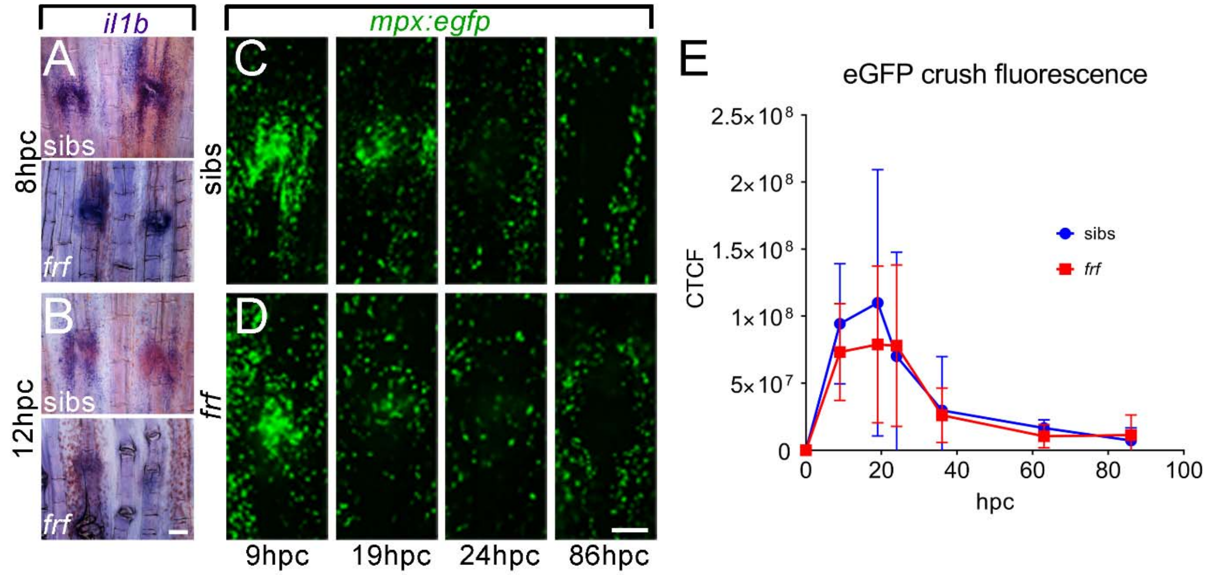

Figure S2: OI fractures mount a wild-type inflammation response

A-B: Images of fractured fin rays in sibling and *frf* adults stained by in situ hybridisation for *il1b,* at 8 hours post crush (hpc) (A) and 12hpc (B). No difference in leukocyte recruitment was observed. **C-E:** Confocal images of neutrophils labelled with the *mpx:egfp* transgene in induced fractures of *frf* (D) or siblings (C) at stated hours post crush (hpc). The extent of eGFP fluorescence at the fracture over the repair process was quantified in ImageJ and is presented (E). There is no measurable change in fluorescence between WT and *frf*. E: n=8 per point. ANOVA with Sidak post test. Scale Bar B=100µm.

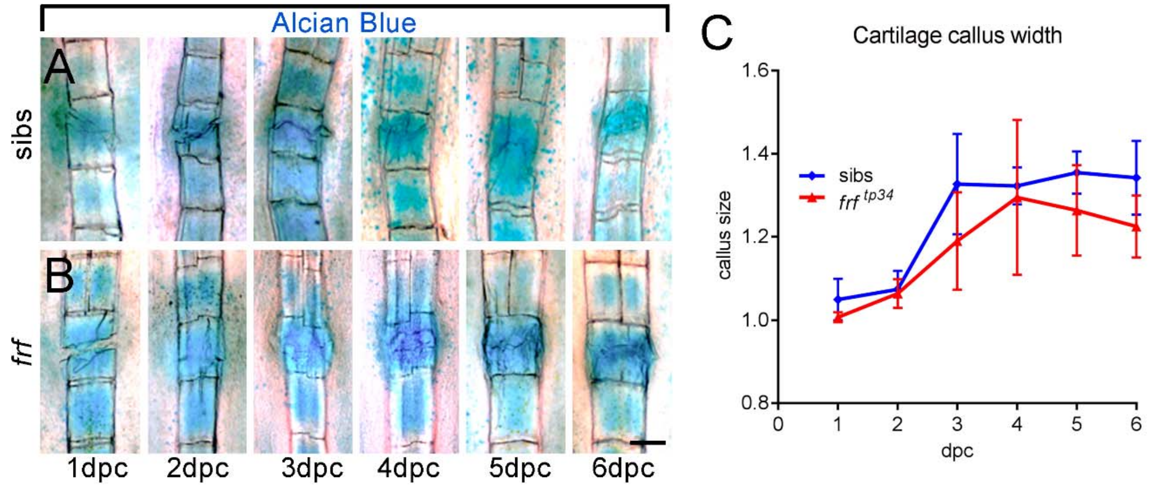

Figure S3: Cartilage callus formation is not affected in induced fractures in an OI model.

A-B: Alcian Blue staining of induced fin ray fractures in *frf* (B) compared to siblings (A) at given days post crush (dpc). Plotting the relative width of the Alcian blue callus (by dividing the callus width by the adjacent unfractured ray width) showed no statistical difference to the sibling controls at any stage of repair (C). C: n=4 per point. ANOVA with Sidak post test. Scale Bar B=100μm.

Disease Models & Mechanisms • Supplementary information

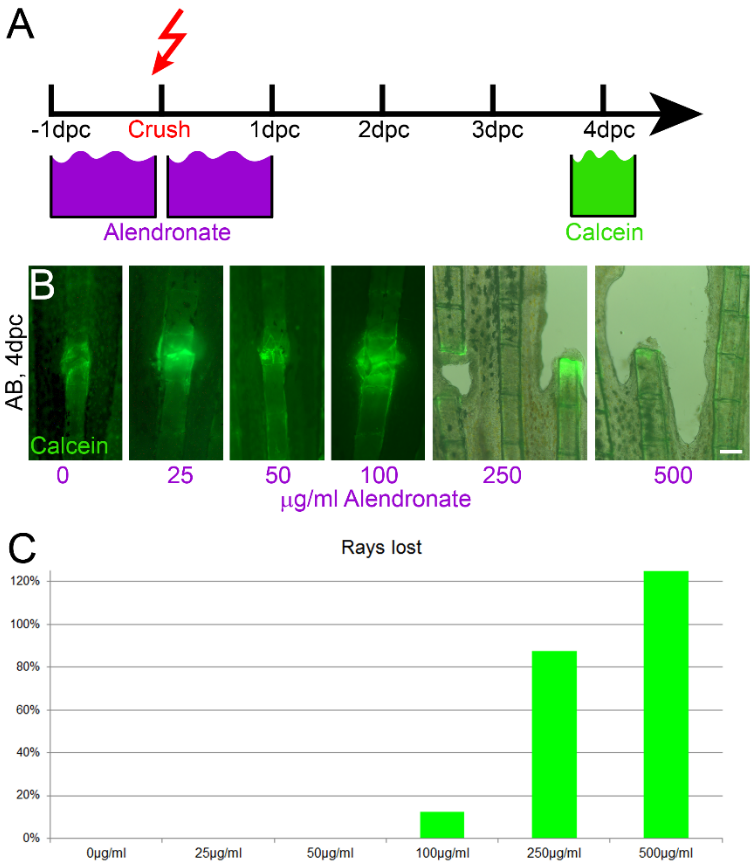

Figure S4: Extended exposure to high doses of alendronate results in loss of fin rays following fracture

A: Experiment outline of exposure to alendronate before and after fracture, followed by visualisation following Calcein staining. **B:** Images of fin ray fractures stained fluorescently with Calcein at 4dpc. Images of fins exposed to higher concentrations are shown with brightfield. Up to 100µg/ml there was no detriment to callus formation, however at higher concentrations, fin rays were often lost distal to the fracture or fractures remained disjoined. The proportion of lost rays for each concentration of alendronate is presented in (C). Often adjacent unfractured rays would also be lost, hence percentage of rays lost is higher than 100% for the 500µg/ml concentration. C: n=8 per point. Scale Bar B=100µm.

Disease Models & Mechanisms • Supplementary information

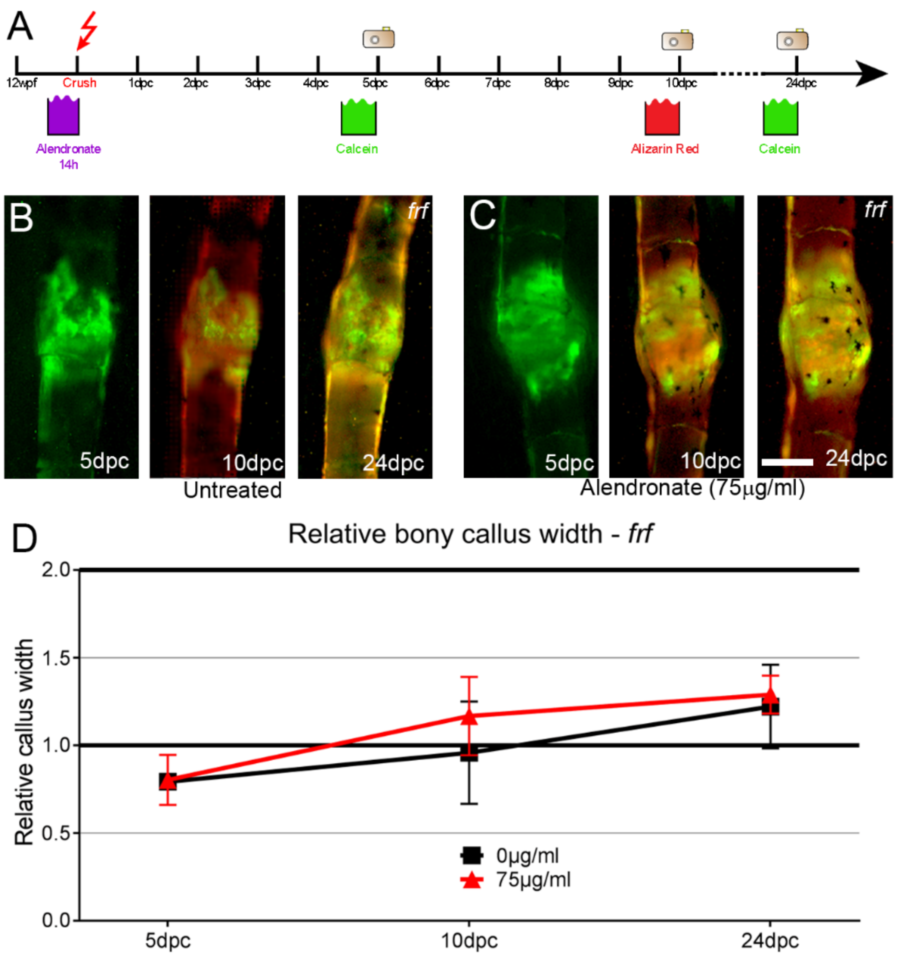

Figure S5: Single pulse exposure to alendronate does not alter callus growth in *frf*
A: Outline of experimental regime for single 14h pulse exposure of *frf* to 75μg/ml alendronate, fracturing and subsequent imaging of fins following alternate staining with Calcein (green) or Alizarin Red (red) to visualise callus growth. **B-C:** Fluorescent images of *frf* fractures either treated with 75μg/ml alendronate (C) or untreated (B), and then imaged for Calcein and Alizarin Red at days post fracture (dpc). The relative bone callus width was compared, but no difference in callus growth was noted upon pulse treatment with alendronate (D), as seen with cyclical exposure. D: n=8 per point. ANOVA with Sidak post test. Scale Bar C=100μm.

Disease Models & Mechanisms • Supplementary information

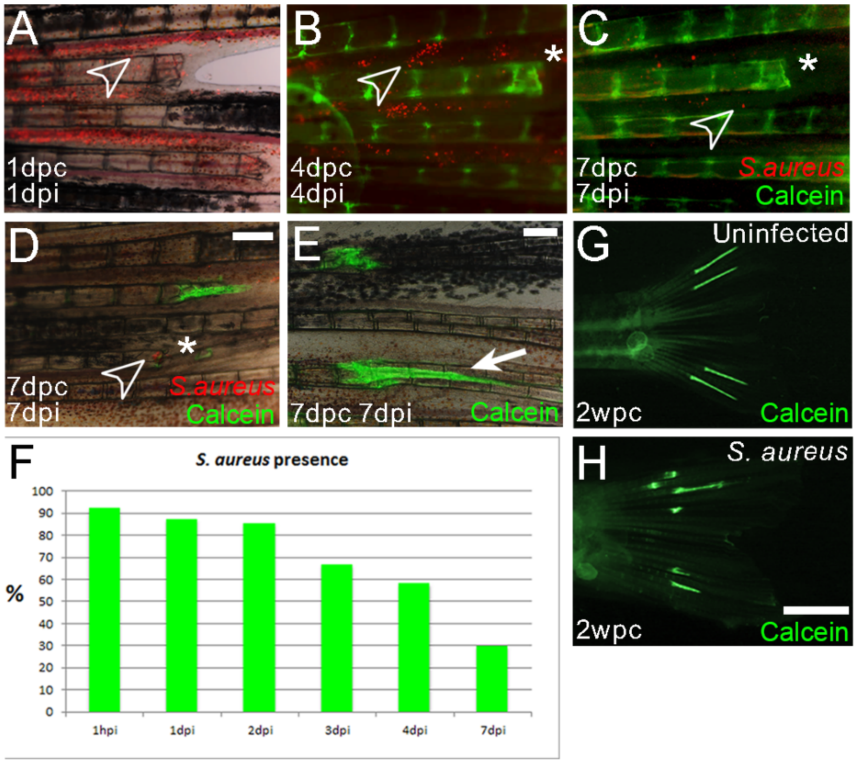

Figure S6: Injection of high volumes of *S. aureus* into fracture sites lead to prolonged infection but loss of distal rays and inhibition of regeneration.
A-F: Images of fractured fin rays injected with 4nl mCherry expressing *S. aureus* (red; A-D) and shown with brightfield view (A, D) and/or stained with Calcein to show the bone regrowth (green; B-E). Images are taken at 1 (A), 4 (B) or 7 (C-E) days following fracture and infection. Injecting this volume following fracture resulted in almost immediate loss of the distal ray (A), yet retention of visible bacteria up to 7dpi (F). Retained bacteria (Arrowheads) led to cessation of regeneration (Asterisks; B, C, D) or misaligned regrowth such that the regenerating ray invades an adjacent unaffected ray (Arrow; E). **G-H:** Fluorescent images of uninfected (G) or *S. aureus* infected (G) fins 2 weeks post crush, which were lost following infection. Infection additionally caused spontaneous fractures in adjacent fin rays. Even when bacteria had cleared, impaired recovery was still evident due to prior infection (G), where regrowth (strongly labelled by Calcein) was impeded compared to uninfected (F). F: n=40 per point. Scale Bar D=200µm; E=300µm; H=2mm.

Disease Models & Mechanisms • Supplementary information

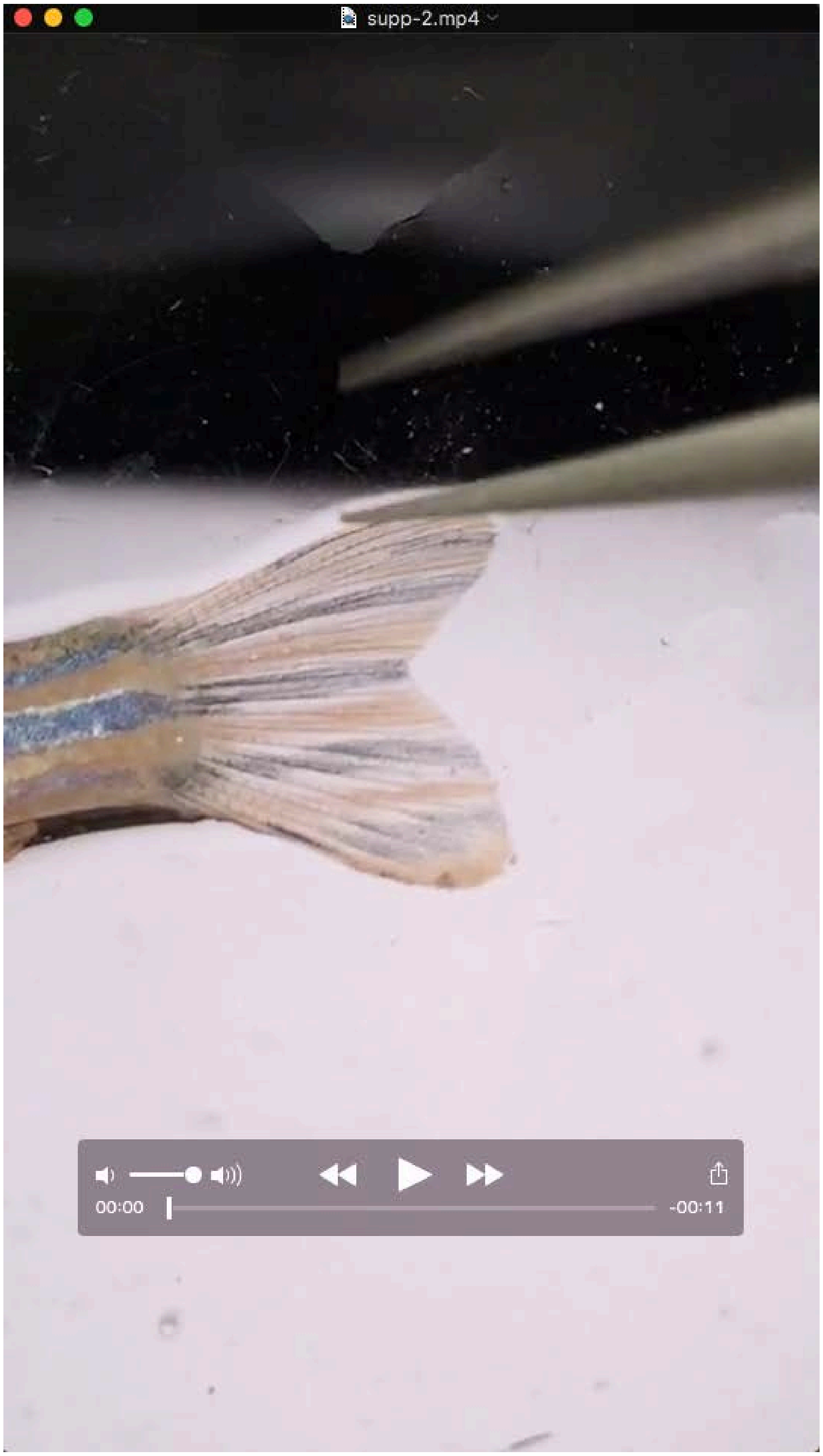

Movie 1: Rapid crush fracturing of zebrafish lepidotrichia

Crush fractures are made in four lepidotrichia per tail fin of a zebrafish adult using forceps under a dissecting microscope, targeting the bone segment just anterior to the first bifurcation. The method is reproducible and rapid.

Disease Models & Mechanisms • Supplementary information
